# Rapid Detection of Multiple Classes of β-Lactam Antibiotics in Blood Using an NDM-1 Biosensing Assay

**DOI:** 10.3390/antibiotics10091110

**Published:** 2021-09-14

**Authors:** Qinglai Meng, Yao Wang, Yali Long, Aiping Yue, Michael Mecklenburg, Shuaiyan Tian, Yujia Fu, Xiangyu Yao, Jianyi Liu, Dewei Song, Changxin Wu, Bin Xie

**Affiliations:** 1 The Key Laboratory of Chemical Biology and Molecular Engineering of National Ministry of Education, Shanxi Provincial Key Laboratory of Medical Molecular Cell Biology, Institute of Biomedical Sciences, Shanxi University, Taiyuan 030006, China; 201923105005@email.sxu.edu.cn (Y.W.); tianshuaiyan123@163.com (S.T.); FuYuJiaF@163.com (Y.F.); 202023105014@email.sxu.edu.cn (X.Y.); 2 Hospital of Shanxi University, Shanxi University, Taiyuan 030006, China; longyali@sxu.edu.cn (Y.L.); yueap@email.sxu.edu.cn (A.Y.); 3 Omik Bioscience AB, SE-22363 Lund, Sweden; michaelmecklenburg@gmail.com; 4 Division of Chemical Metrology and Analytical Science, National Institute of Metrology, Beijing 100029, China; liujy@nim.ac.cn; 5 Pure and Applied Biochemistry, Department of Chemistry, Lund University, SE-22100 Lund, Sweden

**Keywords:** β-lactam antibiotics, therapeutic drug monitoring, critically ill patients, thermometric biosensor, NDM-1

## Abstract

Currently, assays for rapid therapeutic drug monitoring (TDM) of β-lactam antibiotics in blood, which might be of benefit in optimizing doses for treatment of critically ill patients, remain challenging. Previously, we developed an assay for determining the penicillin-class antibiotics in blood using a thermometric penicillinase biosensor. The assay eliminates sample pretreatment, which makes it possible to perform semicontinuous penicillin determinations in blood. However, penicillinase has a narrow substrate specificity, which makes it unsuitable for detecting other classes of β-lactam antibiotics, such as cephalosporins and carbapenems. In order to assay these classes of clinically useful antibiotics, a novel biosensor was developed using New Delhi metallo-β-lactamase-1 (NDM-1) as the biological recognition layer. NDM-1 has a broad specificity range and is capable of hydrolyzing all classes of β-lactam antibiotics in high efficacy with the exception of monobactams. In this study, we demonstrated that the NDM-1 biosensor was able to quantify multiple classes of β-lactam antibiotics in blood plasma at concentrations ranging from 6.25 mg/L or 12.5 mg/L to 200 mg/L, which covered the therapeutic concentration windows of the tested antibiotics used to treat critically ill patients. The detection of ceftazidime and meropenem was not affected by the presence of the β-lactamase inhibitors avibactam and vaborbactam, respectively. Furthermore, both free and protein-bound β-lactams present in the antibiotic-spiked plasma samples were detected by the NDM-1 biosensor. These results indicated that the NDM-1 biosensor is a promising technique for rapid TDM of total β-lactam antibiotics present in the blood of critically ill patients.

## 1. Introduction

The optimization of antibiotic dosing regimens for patients with antimicrobial-resistant (AMR) infections has the potential to dramatically improve therapeutic outcomes while reducing the development of antibiotic resistance. This is especially important when treating critically ill patients where administration of standard antibiotic dosages sometimes results in subtherapeutic antibiotic blood concentration levels that adversely affect treatment outcomes [1,2]. This is caused by pathophysiological changes that disturb the pharmacokinetics (PK) of antibiotic distribution, degradation and clearance. High intra- and interpatient PK variability among critically ill patients makes determining the optimal antibiotic dosage for any given patient challenging [3,4,5]. Predictors have been identified that make it possible to identify at-risk patients that require more individualized dosing regimens [6]. One approach for addressing PK variability among critically ill patients undergoing treatment involves therapeutic drug monitoring (TDM) [7]. Optimization of blood drug levels using TDM has been successfully used to improve patient outcomes for a variety of illnesses [8,9]. This strategy has also been shown to improve outcomes for critically ill patients undergoing antibiotic treatment [10].

Currently, β-lactam antibiotics are the most widely used group of antimicrobial agents due to their safety, efficacy and broad spectrum [11]. The β-lactam antibiotics are divided into four classes: penicillins, cephalosporins, carbapenems and monobactams [12]. They have been widely used and are considered to be safe and effective for treating a range of infectious diseases. However, the safety of antibiotics is increasingly being challenged due to mounting evidence that elevated β-lactam concentrations can have neurotoxic/nephrotoxic effects, while subtherapeutic antibiotic blood concentrations reduce the treatment efficacy [13,14,15]. Consequently, TDM is increasingly being implemented in order to prevent over- and underdosing, thereby improving treatment efficacy whilst minimizing toxic side effects.

Unlike routine monitoring of aminoglycoside and glycopeptide antibiotics using commercial assays, the methods used to monitor β-lactam antibiotics have been largely developed in-house [16]. Among these, liquid chromatographic (LC) or mass spectrometric (MS) detection are the most widely used methods for determining β-lactam antibiotic concentrations in serum or plasma [17,18]. The advantages of these methods include good detection accuracy/stability, wide detection range and simultaneous detection of multiple β-lactam antibiotics/β-lactamase inhibitors (BLIs). However, these methods have drawbacks such as slow and complicated sample preparation, as well as requiring experienced technicians. A number of new strategies for detecting antibiotics are under development, including biomimetic, molecularly imprinted polymers (MIP) and electrochemical-based sensors [19,20]. Each of these strategies has advantages and disadvantages. Some are undergoing clinical trials, but as yet, none have been fully certified.

The Enzyme Thermistor is a flow-injected biosensor device that combines an immobilized enzyme for recognition and catalysis of the substrate with ultrasensitive thermometric detection. The temperature changes generated by the catalytic enthalpy of the enzymes are used to quantify the presence of specific substrates, such as antibiotics [21,22]. Numerous thermometric biosensors have been developed by our group over the past several decades. Detection schemes have been developed that make it possible to rapidly and semicontinuously measure analytes in whole blood, serum and plasma without any sample preparation [23,24,25]. Previously, we developed a penicillinase-based thermal biosensor to rapidly quantitate penicillins in spiked whole blood and serum samples [25]. The elimination of sample preparation and the short measurement cycle time makes our thermal biosensor strategy a promising method for monitoring β-lactam antibiotics in blood [16]. However, the narrow substrate specificity of the penicillinase biological recognition layer limits the biosensor to detecting penicillins. In order to detect other class β-lactam antibiotics, such as cephalosporins and carbapenems, we have developed a novel biosensor based on the New Delhi metallo-β-lactamase-1 (NDM-1). NDM-1 enzyme has broad substrate specificity and is capable of degrading all classes of β-lactam antibiotics with the exception of the monobactams, such as aztreonam. This broad substrate specificity overcomes the inherent limitation of the penicillinase-based biosensor. Here we present results that demonstrate the ability of the NDM-1 biosensor to detect and quantify multiple classes of β-lactam antibiotics in blood plasma in concentration ranges typically seen in critically ill patients. The NDM-1 biosensor detected both free and protein-bound β-lactam antibiotics in the spiked plasma samples. The effect of the β-lactamase inhibitors, such as avibactam (AVI) and vaborbactam, was also investigated. 

## 2. Results

### 2.1. Verification of a Broad Analytical Capacity of the NDM-1 Biosensor 

In order to meet clinical requirements for determination of most therapeutic β-lactam antibiotics, a broad analytical capacity for the most applied antibiotics, in particular cephalosporins and carbapenems, was verified by comparison between the NDM-1 biosensor and the penicillinase biosensor.

**Figure 1:**
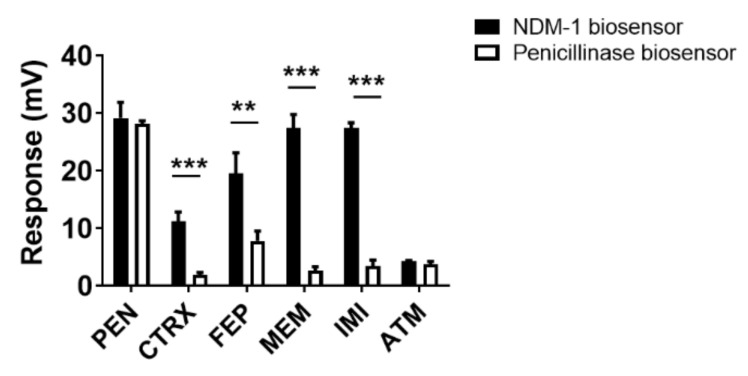
Comparison of hydrolysis of penicillin G, cephalosporins, carbapenems and aztreonam between the NDM-1 biosensor and the penicillinase biosensor. The NDM-1 biosensor and the penicillinase biosensor were loaded with 300 mg/L of penicillin G (PEN), ceftriaxone (CTRX), cefepime (FEP), meropenem (MEM), imipenem (IMI) or aztreonam (ATM), and activity responses of the NDM-1 biosensor and the penicillinase biosensor to the indicated antibiotic are compared and shown. One representative result out of three experiments is shown, *p* < 0.01 and 0.001 are represented as ** and ***, respectively.

Performance in the activity measurement of two cephalosporins (ceftriaxone and cefepime) and two carbapenems (meropenem and imipenem) was assessed and compared using the antibiotics spiked in HEPES buffer while using penicillin G and aztreonam as two references. It was observed that with the antibiotic concentration at 300 mg/L, the NDM-1 biosensor showed significantly greater activity responses to all tested cephalosporins and carbapenems than the penicillinase biosensor except for two comparable activity responses to penicillin G and aztreonam (Figure 1). The results indicated that the NDM-1 biosensor bears a broader analytical capacity than the penicillinase biosensor in the detection of the cephalosporin and carbapenem β-lactam antibiotics.

### 2.2. Determination of Antibiotics in Blood with the NDM-1 Biosensor 

Blood measurement of the β-lactam antibiotics spiked in plasma was studied under two different blood sampling conditions, a heparin sodium tube and an EDTA tube. 

**Figure 2:**
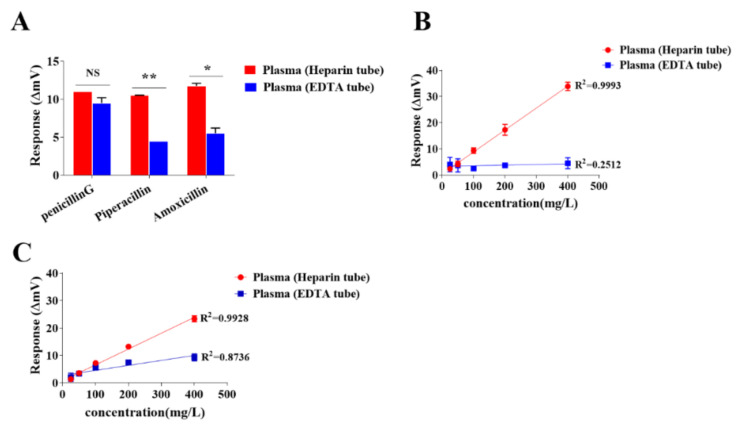
Comparison of activity response of the NDM-1 biosensor to β-lactam antibiotic spiked in plasma collected from heparin sodium tubes and EDTA tubes. Plasma was collected from three healthy donors with heparin sodium tubes and EDTA tubes. Plasma samples prespiked with 100 mg/L of penicillin G, 200 mg/L of piperacillin or 200 mg/L of amoxicillin were subjected to the NDM-1 biosensor, and net activity responses (ΔmV) of the NDM-1 biosensor to the indicated antibiotic in plasma collected from the same donor by heparin sodium tubes and EDTA tubes were compared and shown in panel (**A**). Plasma samples prespiked with 25, 50, 100, 200 and 400 mg/L of meropenem or ceftazidime or with the same volume of buffer were sequentially loaded into the NDM-1 biosensor, and net activity responses (ΔmV) of the NDM-1 biosensor to the each tested concentration of meropenem or ceftazidime in plasma collected by heparin sodium tubes and EDTA tubes were used to generate calibration curves. The calibration curves of plasma meropenem and ceftazidime concentrations detected by the NDM-1 biosensor are shown in panels (**B**,**C**), respectively. The net activity response (ΔmV) of the NDM-1 biosensor to the antibiotic in plasma was calculated by the activity response (mV) of the NDM-1 biosensor to the antibiotic-spiked plasma minus the activity response (mV) of the NDM-1 biosensor to the buffered plasma. *p* < 0.05 and 0.01 are represented as * and **, respectively.

Clinically, plasma samples are usually prepared from whole blood collected by tubes with heparin sodium or EDTA as the anticlog agent. For comparison, the effect of these tubes on the measurements of blood antibiotics is shown in Figure 2. It was observed that among the three tested penicillin antibiotics, there were significantly higher responses using the heparin sodium tubes than the EDTA tubes in the determination of piperacillin-and amoxicillin-spiked plasma samples (Figure 2A). In addition, the response to meropenem-spiked plasma samples showed a linear correlation with the concentration changes using heparin sodium tubes but not EDTA tubes (Figure 2B). Although the linear response of ceftazidime determination was associated with the concentration changes for both heparin sodium tubes and EDTA tubes, higher responses occurred in heparin sodium tubes than in the EDTA tubes (Figure 2C). These data indicated that EDTA could act with a higher inhibitory effect than heparin sodium in the NDM-1 biosensor determinations of β-lactam antibiotics spiked in plasma.

### 2.3. Sensitivity and Measurement Range of Cephalosporins and Carbapenems in Blood Plasma with the NDM-1 Biosensor 

**Figure 3:**
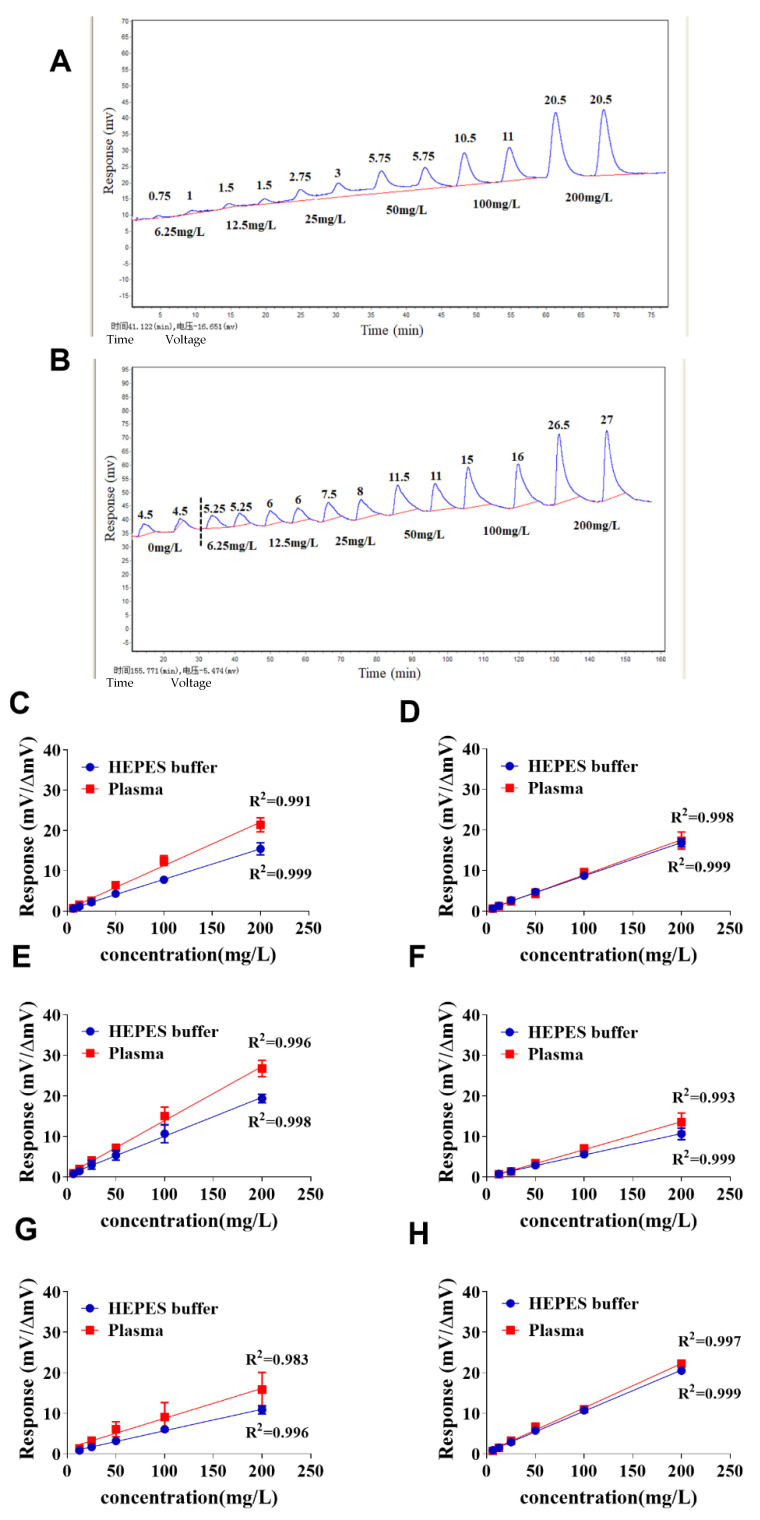
Calibration curves of cephalosporin- and carbapenem-spiked samples. Plasma was collected from three healthy donors with heparin sodium tubes. HEPES buffer or plasma spiked with 6.25, 12.5, 25, 50, 100 and 200 mg/L of imipenem, meropenem, cefaclor, ceftazidime, cefepime or cefoxitin was loaded into the NDM-1 biosensor. The duplicate real responses of the NDM-1 biosensor to each concentration of cefoxitin spiked in HEPES buffer and plasma are shown in panels (**A**,**B**), respectively. The activity responses (mv) of the NDM-1 biosensor to the antibiotic-spiked HEPES buffer and the net activity responses (Δmv) of the NDM-1 biosensor to the antibiotic-spiked plasma were plotted to generate calibration curves, the calibration curves of imipenem, meropenem, cefaclor, ceftazidime, cefepime and cefoxitin are shown in panels (**C**–**H**), respectively. In panels (**F**,**G**), the symbols corresponding to the antibiotic concentration at 6.25 mg/L are not shown because responses were undetectable. The net activity response (ΔmV) of the NDM-1 biosensor to the antibiotic in plasma was calculated by the activity response (mV) of the NDM-1 biosensor to the antibiotic-spiked plasma minus the activity response (mV) of the NDM-1 biosensor to the buffered plasma.

The properties of the NDM-1 biosensor in the determination of carbapenems (imipenem and meropenem); the second, third and fourth generations of cephalosporins (cefaclor, ceftazidime and cefepime); and cephamycins (cefoxitin) in blood plasma were evaluated with the antibiotics spiked in HEPES buffer as control. The real responses of the NDM-1 biosensor to cefoxitin are represented in Figure 3A,B. The results indicated that the linear measurement range was from 6.25 to 200 mg/L for MEM, IMI, CEC and FOX in both plasma and buffer (Figure 3C–E,H) with the sensitivity of 6.25 mg/L, as compared with the measurement range of 12.5 to 200 mg/L and the sensitivity of 12.5 mg/L for CAZ and CEF (Figure 3F,G); The slightly higher responses to the antibiotics spiked in plasma than to the antibiotics spiked in HEPES buffer were observed for the most tested antibiotics (Figure 3C,E–G). This demonstrated that the NDM-1 biosensor was capable of good quantification of cephalosporins and carbapenems in blood with the sensitivity and measurement range necessary in the clinical treatment of critically ill patients with infection, such as septic patients.

### 2.4. Effect of β-Lactamase Inhibitors on the Determinations

**Figure 4:**
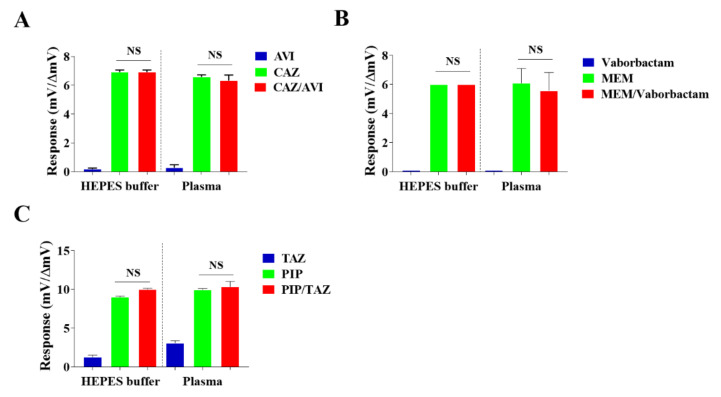
Effect of copresence of β-lactamase inhibitors on detection of β-lactams by the NDM-1 biosensor. Plasma was collected from three healthy donors by heparin sodium tubes. HEPES buffer or plasma samples spiked with 200 mg/L of ceftazidime, 50 mg/L of avibactam or the mixture of them were subjected to the NDM-1 biosensor; the activity responses (mv) of the NDM-1 biosensor to each agent spiked in HEPES and the net activity responses (ΔmV) of the NDM-1 biosensor to each agent spiked in plasma are shown in panel (**A**). HEPES buffer or plasma samples spiked with 100 mg/L of meropenem, 100 mg/L of vaborbactam or the mixture of them were subjected to the NDM-1 biosensor; the activity responses (mV) of the NDM-1 biosensor to each agent spiked in HEPES and the net activity response (ΔmV) of the NDM-1 biosensor to each agent spiked in plasma are shown in panel (**B**). HEPES buffer or plasma samples spiked with 200 mg/L of piperacillin and 50 mg/L of tazobactam or the mixture of them were subjected to the NDM-1 biosensor; the activity responses (mV) of the NDM-1 biosensor to each agent spiked in HEPES and the absolute response activities (ΔmV) of the NDM-1 biosensor to each agent spiked in plasma are shown in panel (**C**). One representative result from three experiments is shown. The net activity response (ΔmV) of the NDM-1 biosensor to the antibiotic in plasma was calculated by the activity response (mV) of the NDM-1 biosensor to the antibiotic-spiked plasma minus the activity response (mV) of the NDM-1 biosensor to the buffered plasma.

Nowadays, some FDA-approved β-lactam antibiotics are coformulated with β-lactamase inhibitors aiming to improve therapeutic efficacy. For instance, piperacillin is coformulated with tazobactam with a mass ratio of 4:1, ceftazidime is coformulated with avibactam with a mass ratio of 4:1 and meropenem is coformulated with vaborbactam with a mass ratio of 1:1 [26]. To evaluate the effect of β-lactamase inhibitors on the determinations of the NDM-1 biosensor to the β-lactam antibiotics, the combined ceftazidime/avibactam antibiotic, the combined meropenem/vaborbactam antibiotic and the combined piperacillin/tazobactam antibiotic were prepared according to the corresponding FDA approved antibiotics. As shown in Figure 4A, the NDM-1 biosensor did not show a significant response to avibactam both in HEPES buffer and in plasma. Meanwhile, the comparable responses of the NDM-1 biosensor to ceftazidime alone and to avibactam combined with ceftazidime both in HEPES buffer and in plasma were observed as well (Figure 4A). Moreover, the comparable responses of the NDM-1 biosensor to meropenem alone and vaborbactam combined with meropenem were observed both in HEPES buffer and in plasma (Figure 4B). In contrast, the NDM-1 biosensor showed apparent activity response to tazobactam both in HEPES buffer and plasma, (Figure 4C). In addition, the tazobactam showed an additive effect on the piperacillin in the buffer. However, the additive effect of the tazobactam on the NDM-1 biosensor in detecting the piperacillin was not observed in plasma (Figure 4C). These data indicated that at the tested concentration, detection of ceftazidime and meropenem by the NDM-1 biosensor was not apparently affected by the copresent avibactam and vaborbactam, respectively, and significant effect of the tazobactam on detection of the NDM-1 biosensor to piperacillin occurred in HEPES buffer but not in plasma.

### 2.5. Effect of Other Antibiotics on Determination of β-Lactam Antibiotic

Multiple lines of evidence support that the combination of one β-lactam antibiotic with another antibiotic exhibits synergism in antimicrobial activity in vitro [27]. Moreover, it was reported that combination therapy using carbapenem with fosfomycin, amikacin or another antibiotic has a synergistic effect on the alleviation of lung inflammation and enhancement of survival [28,29,30,31]. To verify whether combination with fosfomycin or amikacin interferes with the NDM-1 biosensor in the detection of meropenem, activity responses of the NDM-1 biosensor to meropenem alone and meropenem mixed with fosfomycin or amikacin were compared. The combined antibiotics were prepared with mass ratios of meropenem to fosfomycin and amikacin of 1:4 and 1:1, respectively, as reported previously [28,30]. It was observed that within the concentration range from 12.5 to 200 mg/L, the activity responses of the NDM-1 biosensor to fosfomycin or amikacin were very low, and the correlation of the activity responses with the antibiotic concentrations was not significant for either antibiotic (Supplementary Figure S1A). In addition, the activity responses of the NDM-1 biosensor to meropenem alone and meropenem combined with amikacin or fosfomycin were comparable (Supplementary Figure S1B,C). These data indicated that the combined amikacin or fosfomycin did not interfere with the NDM-1 biosensor in the detection of meropenem in buffer, and the NDM-1 biosensor had a good detection specificity.

### 2.6. Study of Plasma Protein-Bound and Unbound β-Lactam Antibiotics

**Figure 5:**
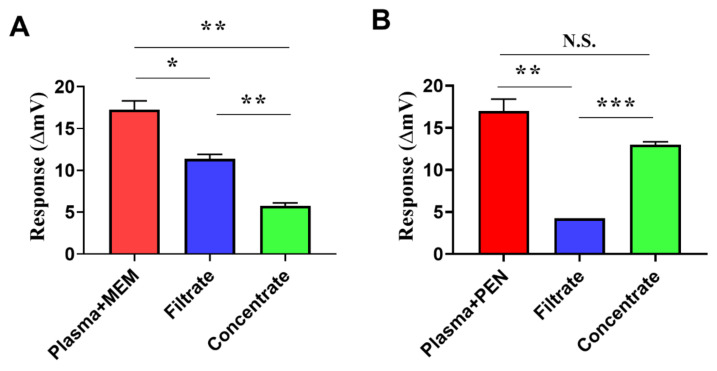
Evaluation of the activity responses of the NDM-1 biosensor to the filtrate and the concentrate from ultrafiltration of β-lactam-spiked plasma. Plasma spiked with 200 mg/L of meropenem, penicillin G or HEPES buffer was processed with ultrafiltration or kept unprocessed. The ultrafiltration of plasma yielded the filtrate and the concentrate, and the concentrate was constituted with HEPES buffer to a volume equivalent to the original volume of the spiked plasma. Then the spiked plasma, the filtrate and the reconstituted concentrate were subjected to the NDM-1 biosensor; the net activity responses (ΔmV) of the NDM-1 biosensor to the meropenem- or penicillin G-spiked plasma, the filtrate and the reconstituted concentrate from ultrafiltration of meropenem- or penicillin G-spiked plasma are shown in panels (**A**) (meropenem-associated data) and (**B**) (penicillin G-associated data), respectively. One representative result from three experiments is shown. The net activity response (ΔmV) of the NDM-1 biosensor to the antibiotic-spiked plasma, the filtrate or the reconstituted concentrate was calculated by the activity response (mV) of the NDM-1 biosensor to the antibiotic-spiked plasma, the filtrate or the reconstituted concentrate generated from ultrafiltration of antibiotic-spiked plasma minus the activity response (mV) of the NDM-1 biosensor to the buffered plasma, the filtrate or the reconstituted concentrate generated from ultrafiltration of the buffered plasma, respectively. *p* < 0.05, 0.01 and 0.001 are represented as *, ** and ***, respectively.

In the blood of patients treated with an antibiotic, the antibiotic exists in protein-bound and unbound (also designated as free) forms, and the proportion of the antibiotic binding on protein depends on the intrinsic property of the antibiotic. In order to identify which antibiotic form the NDM-1 biosensor responded to in the spiked plasma, a study was performed using the NDM-1 biosensor to detect and compare the activity responses to the filtrate and the concentration generated from ultrafiltration of meropenem- or penicillin G-spiked plasma. After ultrafiltration, the protein-bound and unbound forms of the antibiotic are concentrated in the concentration and the filtrate, respectively. The meropenem and penicillin G were reported as having a low activity (2%) and a high activity (65%) to bind plasma protein, respectively [32]. As shown in Figure 5A,B, higher activity responses of the NDM-1 biosensors to the antibiotic-spiked plasma than to its concentrate fraction and to its filtrate fraction were observed to meropenem and penicillin G both. In addition, upon ultrafiltration, there was a greater response of the NDM-1 biosensor to the filtrate fraction than to the concentrate fraction was observed from the meropenem-spiked plasma (Figure 5A), and there was a greater response of the NDM-1 biosensor to the concentrate faction than the filtrate fraction from the penicillin G-spiked plasma (Figure 5B). These data indicated that the measured distributions of meropenem or penicillin G in the concentrate and the filtrate by the NDM-1 biosensor were consistent with the predicted distributions of meropenem or penicillin G in the concentration fraction and the filtrate fraction according to the reported protein binding activity of these antibiotics. Therefore, they also indicated that in antibiotic-spiked plasma, both protein-bound and unbound antibiotics were detected by the NDM-1 biosensor, and the activity response of the NDM-1 biosensor to the antibiotic-spiked plasma originated from the summed activity response of the NDM-1 biosensor to the protein-bound antibiotic and the unbound antibiotic.

## 3. Discussion

Clinically, sufficient PK data points are required for TDM of β-lactam antibiotics for the purpose of optimizing dosage [33]. Therefore, the response time of eligible assays for efficient TDM should be as quick as possible. However, response time for many assays often takes a few days due to practical restrictions and cost limitations [34]. In this context, the thermometric biosensor assay is superior to other conventional assays for TDM of antibiotics due to the fast response time within two to three hours using a flow injection analysis.

In order to cover a broad range of antibiotics applied in therapeutics, a versatile β-lactamase that is capable of hydrolysis of most classes of β-lactam antibiotics was employed in the thermometric biosensor assay. As compared with our previous penicillinase-based biosensor assay for detection of limited types of the penicillin-class antibiotics in blood [25], NDM-1 was much more powerful and efficient for catalyzing β-lactam antibiotics as NDM-1 is a metallo-β-lactamase carbapenemase exhibiting potent hydrolytic activity towards three classes of β-lactam antibiotics, namely penicillins, cephalosporins and carbapenems [35]. As expected, the NDM-1 biosensor showed significantly improved catalytic activity and capacity for the detection of ceftriaxone, cefepime, imipenem and meropenem (Figure 1). In addition, the higher activity response to meropenem and imipenem than ceftriaxone and cefepime, the poor activity response to aztreonam (Figure 1) and the higher response to the antibiotics spiked in the plasma collected from heparin tubes than from EDTA tubes (Figure 2) are consistent with the NDM-1 enzymatic characteristics reported previously [36,37]. These results indicated that the NDM-1 biosensor assay well demonstrated the enzymatic characteristics of NDM-1, thus showing promise in TDM of cephalosporins and carbapenems.

As the β-lactam antibiotics are time-dependent antimicrobial agents, the duration that the free (protein unbound) antibiotic concentration is greater than the minimal inhibitory concentration (MIC) (*f* T_>MIC_) determines microbicidal efficacy [38]. For critically ill patients, the recommended pharmacodynamic (PD) target for the β-lactams is at least 100% *f* T_>MIC_ for the given pathogen [39]. To eliminate the pathogens in maximal efficacy, trough free antibiotic concentrations at 4–5 times the MIC of the targeted pathogen (50–100% *f* T_>4–5 × MIC_) were chosen for patients with severe infection or continuous renal replacement therapy [40,41]. Therefore, according to guidelines from the European Committee on Antimicrobial Susceptibility Testing (EUCAST) and the French Society of Pharmacology and Therapeutics, in the setting of critically ill patients, protein unbound concentrations of the tested cephalosporins (cefaclor, ceftazidime, cefepime and cefoxitin) and carbapenems (meropenem and imipenem) in plasma during trough (steady-state) phase after intermittent administration of β-lactams should be maintained above 16 and 8 mg/L, respectively [26,42]. Given that the target total antibiotic concentration is equivalent to or greater than the target free antibiotic concentration depending on the protein binding rate of the tested antibiotic, the target total concentrations of the tested four cephalosporins and two carbapenems should be ≥16 and 8 mg/L, respectively. The NDM-1 biosensor assay had a broad range for quantitating β-lactams in the spiked plasma. Actually, the upper limit of quantification (ULOQ) of the NDM-1 biosensor for all the tested antibiotics was up to 1000 mg/L (data not shown). Because the maximal concentrations (*C*_max_) of almost all β-lactam antibiotics in plasma are below 200 mg/L after antibiotic administration, 200 mg/L was set up as the upper concentration of the calibration curve of the tested antibiotics in this study. Since the lower limits of quantification (LLOQs) of the NDM-1 biosensor were 6.25 mg/L for detecting cefaclor, cefoxitin, meropenem and imipenem and 12.5 mg/L for detecting ceftazidime and cefepime in the spiked plasma (Figure 3) and the NDM-1 biosensor was demonstrated to detect a total concentration of β-lactam antibiotics in plasma (Figure 5), the NDM-1 biosensor should be eligible for TDM of β-lactam antibiotics even in the case of rapid drawing of a PK curve of the tested β-lactam antibiotic in the treated critically ill patients with 4–5 × MIC as the target therapeutic concentration.

To avoid hydrolysis by the β-lactamases produced in the antimicrobial-resistant bacteria, some β-lactam antibiotics are produced by coformulation with β-lactamase inhibitors (BLIs), which are classified as the β-lactam inhibitors such as sulbactam and tazobactam, the diazabicyclooctane (DBO) inhibitors such as avibactam [11] and the boronic acid inhibitors such as vaborbactam. Among FDA-approved BLIs, most are β-lactam inhibitors; avibactam and vaborbactam are two FDA-approved inhibitors other than the β-lactam class [11]. Functionally, avibactam exhibits potent inhibitory activity on class A, class C and some class D β-lactamases (OXA-48-like carbapenemases) and has no effect on metallo-carbapenemases [11]. Similarly, vaborbactam was demonstrated to inhibit class A and C β-lactamases but poorly inhibited metallo-carbapenemases and OXA-48-like carbapenemases [43,44]. Therefore, the NDM-1 carbapenemase, as a metal-ion-dependent carbapenemase, is inhibited by a metal ion chelator such as EDTA, rather than by avibactam, vaborbactam or tazobactam. However, these inhibitors themselves may interfere with the sensing process of NDM-1 and thus affect measurements of coformulated β-lactam antibiotics. Therefore, the effects of the NDM-1 biosensor responses to the β-lactam antibiotics in the presence or absence of three different coformulated inhibitors were evaluated. At the tested concentration, which was much higher than the maximal concentration of the tested inhibitor in plasma from administrated patients, both avibactam and vaborbactam did not show significant effects on the detections of ceftazidime and meropenem, respectively (Figure 4A,B). Moreover, even though there were some effects of tazobactam on detection of piperacillin in HEPES buffer, none were observed in plasma (Figure 4C). The reason for the discrepant effects of the tazobactam on the NDM-1 biosensor in the detection of piperacillin in HEPES and in plasma could be partly due to the dispersion effect of the tazobactam in the different media. Overall, these data support that the NDM-1 biosensor can measure plasma β-lactams coformulated with avibactam and vaborbactam, thus greatly expanding the category of β-lactam antibiotics eligible for TDM by the NDM-1 biosensor.

## 4. Materials and Methods

### 4.1. Chemicals and Reagents

Meropenem (MEM) and ceftazidime (CAZ) were obtained from USP (Rockville, MD, USA). Penicillin G (PEN), cefoxitin (FOX), cefaclor (CEC), ceftriaxone (CTRX), cefepime (FEP) and tazobactam (TAZ) were from Solarbio (Beijing, China). Avibactam (AVI), vaborbactam, fosfomycin (FOS), amikacin (AMK) and imipenem (IMI) were purchased from MedChemExpress (MCE, Monmouth Junction, NJ, USA). The purity of all antibiotics used in this study was higher than 95%. The propylamino-derivatized controlled pore glass (CPG) beads, with a diameter of 125–140 µm and pore size of 50 nm, were obtained from Steinachglas (Steinach, Germany). HEPES was purchased from Sangon Biotech (Shanghai, China). All other chemical reagents were purchased from Sigma-Aldrich (Shanghai, China) unless stated otherwise. Molecular biology enzymes were bought from Takara Bio (Dalian, China), and the primers were purchased from Sangon Biotech (Shanghai, China).

### 4.2. NDM-1 Gene Construct, Protein Expression and Purification

The coding sequence of the full-length NDM-1 gene (GenBank Accession No. JN616388) was synthesized (Sangon Biotech, Shanghai, China). Subsequently, 28 amino acids were removed from the N-terminus and an N-terminal His-6-tag was added to the NDM-1 gene using PCR, and the product was inserted into the expression vector pET-28a (Novagen). The expression and purification of recombinant NDM-1(rNDM-1) were performed as previously described except that the His-6 tag was not removed from the N-terminus of the recombinant enzyme [18]. The purity of rNDM-1 was determined by SDS-PAGE. The hydrolysis of meropenem by the purified recombinant NDM-1 enzyme was analyzed photospectrometrically. Absorbance changes were monitored and used to calculate the activity of the rNDM-1. The purity of the purified rNDM-1 was >95%, and carbapenemase-specific activity of the purified rNDM-1 was confirmed by potent hydrolysis of meropenem in presence of Zn^2+.^ The unit activity of rNDM-1 to hydrolyze meropenem was 108 IU/mg.

### 4.3. Biosensor Preparation

The NDM-1 biosensor consisted of an immobilized NDM-1 enzyme column, a peristaltic pump (Ismatec, Mount Holly, NJ, USA) connecting with an injection valve with a 350 µL sample volume and the Enzyme Thermistor instrument (Omik Bioscience AB, Lund, Sweden). The working temperature was set to 30 °C, and the operational flow rate was 10.

The NDM-1 column was loaded with 400 µL CPG beads with immobilized rNDM-1 enzyme. The NDM-1-immobilized CPG was prepared using 400 µL CPG beads and 100 units of purified rNDM-1, and the remaining active sites were blocked with 1% BSA prepared with 0.01M PBS for the purpose of reducing background signal from plasma metrics. The penicillinase column was prepared as previously described [19].

### 4.4. Procedure of the NDM-1 Biosensor for Antibiotic Detections

β-Lactam antibiotics used in this study were dissolved with deionized (18.2 MΩ) water to prepare antibiotic stock that was further diluted to the working solutions with 50 mM/L HEPES (150 mM/L of NaCl, 10 µM/L of ZnSO4) at pH 7.5. The HEPES buffer was also used as the running buffer for the biosensor system throughout the study.

Blood samples were collected from healthy volunteers using either heparin sodium tubes or EDTA tubes. Within two hours after collection of the blood, plasma samples were obtained by centrifuge and immediately stored in a −80 °C freezer until experiment.

In the biosensor determination of antibiotic samples, the antibiotic stocks were serially diluted with the HEPES buffer to the defined concentrations and a volume of 450 µL for each sample to be tested. For determination of antibiotic-spiked plasma samples, the antibiotic stocks were firstly serially diluted with the HEPES buffer, followed by mixing with plasma to a series of defined concentrations of the testing antibiotic samples, including a HEPES buffer-spiked plasma sample with blank antibiotic as a negative control. The net value of the measured antibiotic concentration was recalibrated by deduction of the control signal from the total measurement signal.

### 4.5. Preparation and Measurement of Ultrafiltrated Blood Plasma Samples

Four hundred fifty microliters of plasma samples spiked with antibiotics or buffer was loaded into the upper chamber of an Amicon Ultra-0.5 mL 30 KD cut-off centrifugal filter device (Millipore, Darmstadt, Germany) for centrifugation for 10 min at 14,000× *g* to obtain the filtrate fraction in the lower chamber and the concentrate fraction in the upper chamber. To determine antibiotic distribution in the filtrate fraction and the concentrate fraction, the filtrate fraction was directly analyzed by the NDM-1 biosensor, and the concentrate fraction was reconstituted (diluted) with the running buffer to 450 μL before analysis by the NDM-1 biosensor. The net activity response (Δmv) of the NDM-1 biosensor to the spiked antibiotic in the concentrate fraction was calculated as (the activity response to the reconstituted concentration fraction from the antibiotic-spiked plasma — the activity response of the reconstituted concentration fraction from the buffered plasma) × dilution factor.

### 4.6. Statistical Analysis

Statistical analysis was performed with Prism 8.0 (GraphPad Software Inc., San Diego, CA, USA). Unpaired Student’s *t*-test was used to compare differences throughout the study. All tests were considered statistically significant at *p* < 0.05.

## 5. Conclusions

In this study, we have developed a novel thermometric NDM-1 biosensing assay for rapid and quantitative measurements of total cephalosporin and carbapenem β-lactam antibiotics in blood without the need for any pretreatment of samples in order to reduce the measurement cycle period to within 2–3 h. A broad detection range to cover therapeutic windows of most β-lactam antibiotics for the treatment of critically ill patients was achieved using the NDM-1 as catalysis for thermometric biosensor, which allows for a routine TDM of β-lactam antibiotics in the clinical setting for critically ill patients. In addition, a potential application in TDM of original cephalosporins and carbapenems, as well as BLIs coformulated with β-lactam antibiotics, in blood plasma was evaluated. The data demonstrated that the NDM-1 biosensor is suitable for the rapid detection of plasma totals of multiple classes of β-lactam antibiotics with high sensitivity, speed and efficiency.

## Supplementary Materials

The following are available online at https://www.mdpi.com/article/10.3390/antibiotics10091110/s1, Figure S1: Effect of the combined amikacin or fosfomycin on detection of meropenem by the NDM-1 biosensor.
