# Supplementary figures and images for: Rapid Detection of Multiple Classes of β-Lactam Antibiotics in Blood Using an NDM-1 Biosensing Assay
Source: Antibiotics (Basel). 2021 Sep 14;10(9):1110. doi: 10.3390/antibiotics10091110 (PMC8468087; doi:10.3390/antibiotics10091110)

**A**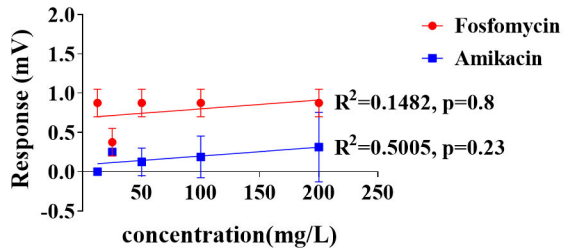**B**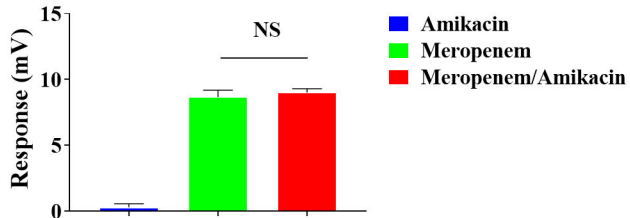**C**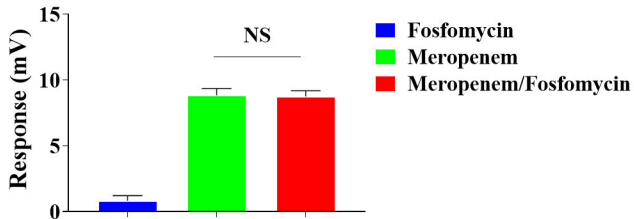

Supplement: Supplementary file 1 [file antibiotics-10-01110-s001.zip › antibiotics-1350897-supplementary.pdf]
